# **Does SUFU’s best practice policy statement regarding antibiotic prophylaxis predict urinary tract infection after urodynamic study?** **Evidence from a retrospective cohort**

**DOI:** 10.1007/s00345-025-05979-6

**Published:** 2025-10-04

**Authors:** Omri Schwarztuch Gildor, Elad Yosef, Netanel Levin, Anna Itshak, Rony Vainrib, Michael Vainrib

**Affiliations:** 1 Department of Urology, Meir Medical Center, 59 Tchernichovsky st, Kfar-Saba, 4428164 Israel; 2 Faculty of Medical and Health Sciences, Tel Aviv University, Tel Aviv, Israel; 3 Faculty of Medicine, The Hebrew University of Jerusalem, Jerusalem, Israel

**Keywords:** Urodynamics, UTI, Antibiotic prophylaxis

## Abstract

**Purpose:**

To validate the Society of Urodynamics, Female Pelvic Medicine & Urogenital Reconstruction (SUFU) Best Practice Policy Statement (BPPS) risk factors (RFs) for predicting urinary-tract infection (UTI) after urodynamic study (UDS) and to identify possible RFs that could better guide antibiotic prophylaxis.

**Methods:**

A retrospective cohort study included all adults undergoing UDS at a single institution. Patients with asymptomatic bacteriuria received a 3-day antibiotic course, while those without bacteriuria received no prophylaxis. The primary endpoint was a culture-confirmed UTI within 7 days. Regression analysis was performed to check SUFU BPPS RFs as predictors for post-UDS UTI.

**Results:**

Among 1666 patients (median age 68 years; 42% female), UTI occurred in 31 (1.9%). Abnormal GU anatomy was found as a predictor for post-UDS UTI (OR = 3.26, *p* = 0.033). Other examined RFs were not found to predict post-UDS UTI. More concise variables were identified as statistically significant predictors: hydronephrosis (OR = 4.98, *p* = 0.004), elevated post-void residual (PVR) (OR = 2.80, *p* = 0.011), and NLUTD due to neurologic disease (OR = 2.27, *p* = 0.042). In multivariate analysis, elevated PVR and NLUTD caused by neurologic disease remained independent predictors.

**Conclusion:**

The current SUFU BPPS criteria exhibit limited accuracy for predicting post-UDS UTI. Our study emphasizes hydronephrosis, NLUTD caused by neurologic disease, and elevated PVR as predictors of post-UDS UTI. Updating prophylactic guidelines to incorporate these findings could enhance patient safety and antimicrobial stewardship without compromising infection control.

## Introduction

Urodynamic studies (UDS) are common diagnostic procedures used to assess lower urinary tract function in patients with voiding dysfunction, incontinence, or other urological conditions. While generally safe, UDS pose a risk of iatrogenic urinary tract infections (UTIs), with reported rates from 1% to 6%, depending on patient characteristics and clinical protocols. Recognizing patients at increased risk for UTI is essential for guiding the use of prophylactic antibiotics (AP) and minimizing unnecessary antimicrobial exposure [1–5].

To address this, the Society of Urodynamics, Female Pelvic Medicine & Urogenital Reconstruction (SUFU) published the Best Practice Policy Statement (BPPS) in 2012 that recommends AP in “non-indexed” patients [6]. This was further clarified in their 2017 update, which recommended routine AP for patients at high risk of post-procedural UTI [7]. These patients include those with untreated asymptomatic bacteriuria (ASB), individuals using clean intermittent catheterization (CIC), those with neurogenic lower urinary tract dysfunction (NLUTD), those who use an indwelling catheter, older adults (aged 70 and above), those with bladder outlet obstruction, elevated post-void residuals (PVR), orthopedic implants, and individuals with compromised immunity. SUFU recommends a single oral dose of antibiotics, typically trimethoprim-sulfamethoxazole or ciprofloxacin, administered 30–60 min before the UDS. Most of the recommendations have a low level of evidence (III–IV).

Despite the widespread adoption of these recommendations, the predictive validity of SUFU criteria for post-UDS UTI hasn’t been independently validated in a large real-world cohort.

In this study, we aim to assess the performance of SUFU BPPS risk criteria in predicting UTI after UDS. Using a large, single-institution retrospective database, we evaluate the association between guideline-defined risk factors (RF) and the development of UTI. Our goal is to provide empirical evidence to support or refine existing prophylaxis guidelines in the context of antimicrobial stewardship.

## Methods

In our institution, we use a urine culture (UC) based protocol before UDS. All patients are required to undergo a UC before UDS. AP wasn’t administered before UDS unless ASB was present. A positive UC was defined as having ≥ 10^5^ CFU/mL of a single organism. The same threshold was applied to all patients and genders. In such cases, patients received a three-day course of culture-guided oral antibiotics before UDS. UCs with multiple organisms were classified as mixed flora. In these cases, a single dose of ciprofloxacin was given one hour before UDS. A previous study reported a similar post-UDS UTI rate, accompanied by a significant reduction in AP usage, which enables us to evaluate SUFU RFs retrospectively [8].

### Study design and population

We conducted a retrospective cohort study including all adult patients who underwent UDS at our institution between October 2013 and December 2023.

There are no exclusion criteria in this study.

### Procedure protocol

All procedures were performed by a single fellowship-trained urologist using a Laborie air-charged urodynamic system. All UDS procedures followed a standardized technique. A 7-French urethral catheter was inserted under sterile conditions. New sterile saline or contrast media and pump tubing were used for each patient. A rectal catheter was placed. The UDS was conducted under fluoroscopic guidance (video UDS), with the addition of electromyography (EMG) patches when clinically indicated.

### Risk factors assessment

SUFU BPPS-defined RFs were retrospectively extracted from medical records. They included: ASB, NLUTD, post-void residual (PVR), and the use of an indwelling catheter or CIC. SUFU recommends avoiding AP in patients with presumed normal genitourinary (GU) anatomy but doesn’t provide specific recommendations for patients with known hydronephrosis and/or bladder diverticula. Presuming they are considered abnormal GU anatomy and result from lower urinary tract dysfunction, we also took them as RFs. UDS reports were reviewed, and the following data were extracted from the pressure-flow study: maximal flow rate (Qmax) and detrusor pressure at Qmax (Pdet@Qmax). A PVR > 200mL was considered significant. Bladder outlet obstruction (BOO) was defined as a BOOI > 40 in men. In women, BOO was defined by a combination of Qmax < 10 mL/s and Pdet@Qmax > 30cmH_2_O. Data regarding immunosuppression, corticosteroids, inherent immune deficiency (recommendation #15), and orthopedic implants (recommendation #19) weren’t available. Therefore, according to SUFU recommendations, up to six possible RFs were evaluated for each patient in this study (Table 1).

**Table 1:** Examined SUFU BPPS risk factors

SUFU BPPS recommendation number	Risk factor	Included patients
4	Abnormal GU anatomy	At least one of the following: - Hydronephrosis - Bladder diverticula
5	NLUTD	Lower urinary tract dysfunction caused by at least one of the following: - Neurologic Disease - Spinal cord injury
6	BOO and/or elevated	At least one of the following: - BOOI > 40 in men, or combination of Qmax < 10 mL/s and Pdet@Qmax > 30 cmH20 in women - PVR > 200 mL
7	Age > 70 y	All patients older than 70 years
12	ASB	Patients with positive urine culture, including patients with mixed flora
16	Indwelling Catheter or CIC	All patients with indwelling catheters or performing CIC
15	Immunosuppression	Information is not available. not evaluated in the study
19	Orthopedic implants	Information is not available. not evaluated in the study

### Outcome definition

The primary outcome was UTI within 7 days following UDS, defined as the presence of urinary symptoms (e.g., dysuria, urgency, frequency, suprapubic pain, or fever) confirmed by a positive UC.

### Ethics considerations

The Institutional Review Board (IRB) reviewed and approved the study protocol (Meir Medical Center – Helsinki Committee, MMC-0182-23). Given the study’s retrospective nature, the IRB issued a waiver of informed consent. All data were anonymized to ensure patient confidentiality and compliance with data protection regulations.

### Statistical analysis

Descriptive statistics were used to summarize baseline demographic and clinical characteristics. Continuous variables were reported as median and interquartile range (IQR) and compared using the Mann–Whitney U test. Categorical variables were summarized as frequencies and percentages and compared using the chi-square test or Fisher’s exact test, as appropriate.

Binary logistic regression was performed to identify independent associations between SUFU RFs and post-UDS UTI. Adjusted odds ratios (ORs) and 95% confidence intervals (CI) are reported. A two-sided p-value < 0.05 was considered statistically significant. All analyses were conducted using SPSS version 29 (IBM Corp., Armonk, NY, USA).

## Results

A total of 1,666 patients underwent UDS during the study period. The median age was 68 (IQR 56–76), and 42.4% were female. Among the cohort, 352 (21.1%) patients had ASB and received AP before the procedure. The remaining 1,314 (78.9%) patients didn’t receive AP. 46% of men had benign prostatic enlargement (prostate > 30 g), and 489 (29.4%) had NLUTD due to spinal cord injury and/or neurologic disease. The most common neurologic diseases were cerebrovascular disease, diabetic neuropathy, multiple sclerosis, and Parkinson’s disease. According to SUFU, 279 (16.7%) patients without any RF are classified as “index” patients, and the remaining 1,387 (83.3%) patients are “non-index” patients who should have received AP. The prevalence of each SUFU RF and their distribution between ASB and negative UC are shown in Table 2. All studied SUFU RFs, except BOO and/or elevated PVR, were statistically significantly more prevalent in patients with ASB. The primary outcome of post-UDS UTI was observed in 31 (1.9%) patients. No statistically significant difference was found between the ASB group and the negative UC group (1.7% vs. 1.9%, respectively, *p* = 0.807).

**Table 2:** Risk factors and outcome measurements

Risk Factor (SUFU recommendation) *n*, (%)	ASB (12) *N* = 352	Negative urine culture *N* = 1,314	*p*-value	All Patients *N* = 1,666
Abnormal GU anatomy (4) - Hydronephrosis - Bladder diverticula	27 (7.7%) 22 (6.3%) 7 (2.0%)	50 (3.8%) 33 (2.5%) 19 (1.4%)	0.002 < 0.001 0.168	81 (4.9%) 55 (3.3%) 26 (1.6%)
Neurogenic LUTD (5) - Neurologic Disease - Spinal cord injury	124 (35.2%) 69 (19.6%) 65 (18.5%)	365 (27.8%) 192 (14.6%) 196 (14.9%)	0.006 0.022 0.104	489 (29.4%) 261 (15.7%) 261 (15.7%)
BOO and/or elevated PVR (6) - BOO - PVR > 200 mL	138 (39.2%) 97 (31.1%) 58 (16.5%)	579 (44.1%) 410 (33.2%) 263 (20.0%)	0.102 0.187 0.135	717 (43.0%) 507 (30.4%) 321 (19.3%)
Age > 70 y (7)	177 (50.3%)	569 (43.3%)	0.019	746 (44.8%)
Patients with chronic catheters (16)	99 (28.2%)	55 (4.2%)	< 0.001	154 (9.2%)
**Post-UDS UTI**	**6 (1.7%)**	**25 (1.9%)**	**0.807**	**31 (1.9%)**

Logistic regression analysis was performed to identify potential predictors of post-UDS UTI (Table 3). All models were adjusted for ASB as a confounder. Analyzing according to the SUFU recommendations, abnormal GU anatomy was found as a statistically significant predictor of post-UDS UTI with OR = 3.26 (1.10–9.62), *p* = 0.003. All other SUFU recommendations weren’t identified as significant predictors of post-UDS UTI. However, when examining specific RFs, hydronephrosis, NLUTD due to neurologic disease, and elevated PVR appeared as potential predictors of post-UDS UTI with OR of 4.98 (1.65–15.1), 2.27 (1.03–4.99), and 2.80 (1.27–6.16), respectively (*p* < 0.05). Age > 70 and patients with indwelling catheters or CIC didn’t reach statistical significance due to p-values of 0.064 and 0.079, respectively.

Two multivariate regression models were developed (Table 4): the SUFU RF model included all SUFU RF recommendations as potential predictors of post-UDS UTI. The concise model was created based on the results of the previous regression analyses and considers hydronephrosis, NLUTD due to neurologic disease, elevated PVR, and age > 70. ASB and chronic catheter use were excluded due to multicollinearity. NLUTD caused by neurologic disease and elevated PVR were significant predictors, with OR of 2.39 (1.07–5.87) and 2.42 (1.06–5.54), respectively. Overall, the concise model demonstrated better performance than the SUFU model.

**Table 3:** Regression models of risk factors

Risk Factor (SUFU recommendation) *n*, (%)	No post-UDS UTI *N* = 1,635	post-UDS UTI *N* = 31	Logistic Regression †	
			O.*R* (95% CI)	*p*-value
Abnormal GU anatomy (4) - Hydronephrosis - Bladder diverticula	73 (4.5%) 51 (3.1%) 26 (1.6%)	4 (12.9%) 4 (12.9%) 0 (0%)	3.26 (1.10–9.62) 4.98 (1.65–15.1) 1.00 (0.00–99.0)	0.033 0.004 0.999
Neurogenic LUTD (5) - Neurologic Disease - Spinal cord injury	476 (29.1%) 252 (15.4%) 256 (15.7%)	13 (41.9%) 9 (29.0%) 5 (16.1%)	1.78 (0.86–3.66) 2.27 (1.03–4.99) 1.04 (0.40–2.74)	0.120 0.042 0.935
BOO and/or elevated PVR (6) - BOO - PVR > 200 mL	700 (42.8%) 496 (32.7%) 310 (19.0%)	17 (54.8%) 11 (35.5%) 11 (35.5%)	1.62 (0.79–3.31) 1.13 (0.54–2.38) 2.80 (1.27–6.16)	0.187 0.747 0.011
Age > 70 y (7)	727 (44.5%)	19 (61.3%)	1.99 (0.96–4.14)	0.064
ASB (12)	346 (21.1%)	6 (19.4%)	0.89 (0.36–2.20)	0.807
Indwelling Catheter or CIC (16)	149 (9.1%)	5 (16.1%)	2.61 (0.90–7.57)	0.079

† Models were adjusted to ASB

**Table 4:** Multivariate regression models for Post-UDS UTI prediction

Risk Factor	All SUFU RFs Model		Concise Model	
	O.*R* (95% CI)	*p*-value	O.*R* (95% CI)	*p*-value
Abnormal GU anatomy (4) - Hydronephrosis - Bladder diverticula	2.95 (0.95–9.16)	0.061	3.10 (0.69–13.9)	0.140
Neurogenic LUTD (5) - Neurologic Disease - Spinal cord injury	1.92 (0.91–4.06)	0.085	2.39 (1.07–5.87)	0.048
BOO and/or elevated PVR (6) - BOO - PVR > 200 mL	1.60 (0.76–3.34)	0.214	2.42 (1.06–5.54)	0.036
Age > 70 y (7)	1.90 (0.90–4.01)	0.090	2.06 (0.89–4.77)	0.091
ASB (12)	0.51 (0.18–1.48)	0.218		
Indwelling Catheter or CIC (16)	1.96 (0.65–5.89)	0.233		
Model summary -2 Log likelihood	287.09		228.53	

## Discussion

In this large, single-center cohort, the incidence of UTI within seven days of UDS was 1.9%, which is at the lower end of the range reported in recent literature [1–5]. Two findings warrant particular emphasis. First, all RFs listed in the SUFU BPPS, except for abnormal GU anatomy, weren’t independently associated with infection in regression modeling. Second, three more specific variables—hydronephrosis, elevated PVR, and NLUTD due to neurologic disease—emerged as dominant predictors, each doubling or quadrupling the odds of post-procedure UTI.

### Asymptomatic bacteriuria

21% of our cohort received AP due to ASB as described earlier. In our analysis, ASB didn’t predict subsequent infection. This aligns with earlier studies examining AP before low-risk procedures, such as cystoscopies [9, 10]. The AUA doesn’t recommend AP for ASB in low-risk procedures unless the patient is pregnant [11]. Snow-Lisy et al. reported a similarly low rate of post-procedure UTI in pediatric patients with ASB, questioning the need for UC before UDS [12]. However, our study is limited in evaluating ASB as a predictor of post-UDS UTI because all of these patients received AP.

### Post-void residual threshold

Elevated PVR has consistently been linked to post-UDS bacteriuria or UTI; however, reported cut-offs range from 50 to 100 mL to the 300 mL threshold endorsed by the AUA’s white paper on chronic urinary retention [13–15]. We used a cut-off of 200 mL as a compromise between sensitivity and specificity. Additionally, we didn’t require at least two measurements over a six-month period to define a patient as having an elevated PVR. The same cut-off was used for men and women, despite the definition of elevated PVR is less clear in women.

### Abnormal GU anatomy

SUFU BPPS doesn’t define “abnormal GU anatomy”. We classified hydronephrosis and bladder diverticula as abnormal, consistent with evidence that obstruction/stasis predisposes to UTI: normal bladders clear bacteria rapidly, whereas obstruction (e.g., BPH, strictures, diverticula, hydronephrosis) promotes infection [16]. In our cohort, diverticula did not predict UTI, but hydronephrosis did, underscoring the need for a clear operational definition in future studies and guidelines.

### Comparison with prior work

Our results generally support a previous validation study by Fox et al. [17], which identified elevated PVR and NLUTD as predictors in 489 patients who underwent UDS without prophylaxis. Notably, 83.4% of their cohort were female, and the rates of hydronephrosis weren’t reported. Additionally, they excluded nearly 30% of their cohort from the analysis due to antibiotic use before UDS. In our study, no patients were excluded. Only 21% received AP, and the use of AP due to ASB was considered a confounder in the regression models.

### Outcome definition and surveillance window

The ideal timeframe for linking infection to UDS is uncertain. Fox et al. used 30 days [17]; we selected 7 days to enhance causal inference. Recent studies support this window and likely explain our lower event rate [12, 18, 19].

### Guideline context

Guidance on AP before UDS is inconsistent. The Canadian Urological Association recommends AP for loosely defined “high-risk” patients [20]. French guidance is more specific: age > 70 and PVR > 200 mL are RFs; AP is optional with a sterile pre-procedure culture, and NLUTD is highlighted as a special subgroup [21]. The AUA advises against AP for routine UDS in healthy, asymptomatic adults [11]. The joint AUA/SUFU NLUTD guideline supports AP for clean-contaminated urologic procedures but gives no clear direction for UDS [22]. Infectious Diseases Society of America (IDSA) guidelines likewise don’t endorse AP for ambulatory urologic procedures [23]. The European Association of Urology discourages routine AP, noting that while it reduces ASB, it hasn’t been shown to lower UTI rates [24]. Overall, these statements reflect a lack of consensus and support a selective, risk-stratified approach rather than universal prophylaxis.

### Strengths and limitations

Strengths include the largest dataset to evaluate SUFU RFs, uniform procedural technique, and strict outcome definition (culture-confirmed UTI within 7 days). Limitations include the retrospective design, mandatory ASB treatment in 21% (which may potentially mask its effect), the possibility of overtreatment with ciprofloxacin in cases with mixed flora, and the lack of data on patients with some BPPS RFs (immunosuppression and orthopedic implants).

## Conclusions

Current SUFU BPPS criteria exhibit limited accuracy in predicting post-UDS UTI. Our study highlights hydronephrosis, NLUTD caused by neurologic disease, and elevated PVR as predictors of post-UDS UTI. An approach that considers specific RFs can reduce the number of patients exposed to AP while ensuring patient safety. Updating prophylactic guidelines to include these findings could improve patient safety and antimicrobial stewardship without undermining infection control.

## Data Availability

No datasets were generated or analysed during the current study.
